# Dr. Leroy D’Etoilles

**Published:** 1860-11

**Authors:** 


DR. LEROY D'ETOILLES, whose name is so honorably connected with lithothrity and diseases of the urinary organs, died recently in Paris.

